# Associations among drinking water quality, dyslipidemia, and cognitive function for older adults in China: evidence from CHARLS

**DOI:** 10.1186/s12877-022-03375-y

**Published:** 2022-08-18

**Authors:** Xi Pan, Ye Luo, Dandan Zhao, Lingling Zhang

**Affiliations:** 1 Department of Sociology, Texas State University, 601 University Drive, San Marcos, TX 78666 USA; 2 Department of Sociology, Anthropology, and Criminal Justice, Clemson University, SC 29634 Clemson, USA; 3 Department of Nursing, University of Massachusetts Boston, MB 02125 Boston, USA

**Keywords:** Drinking water, Dyslipidemia, Cognitive function, Older adults, China

## Abstract

**Background:**

The current study aimed to examine the association between drinking water quality and cognitive function and to identify the direct and indirect effects of drinking water quality and dyslipidemia on cognitive function among older adults in China.

**Methods:**

Primary data for the study were selected from China Health and Retirement Longitudinal Study (CHARLS, 2015) and 4,951 respondents aged 60 and above were included. Data on drinking water quality were selected from the 2015 prefectural water quality data from the Institute of Public and Environment Affairs in China and measured by the Blue City Water Quality Index. Dyslipidemia was measured by self-reported dyslipidemia diagnosis and lipid panel. Three composite measures of cognitive function included mental status, episodic memory, and global cognition. Mixed effects models were conducted to assess the associations between drinking water quality or dyslipidemia and cognitive function. The mediation effects of dyslipidemia were examined by path analyses.

**Results:**

Exposure to high quality drinking water was significantly associated with higher scores in mental status, episodic memory, and global cognition (β = 0.34, *p* < 0.001 for mental status; β = 0.24, *p* < 0.05 for episodic memory; β = 0.58, *p* < 0.01 for global cognition). Respondents who reported dyslipidemia diagnosis had higher scores in the three composite measures of cognitive function (β = 0.39, *p* < 0.001 for mental status; β = 0.27 *p* < 0.05 for episodic memory; β = 0.66, *p* < 0.001 for global cognition). An elevated blood triglycerides was only associated with higher scores in mental status (β = 0.21, *p* < 0.05). Self-reported dyslipidemia diagnosis was a suppressor, which increased the magnitude of the direct effect of drinking water quality on mental status, episodic memory, and global cognition.

**Conclusion:**

Drinking water quality was associated with cognitive function in older Chinese and the relationship was independent of natural or socioeconomic variations in neighborhood environments. Improving drinking water quality could be a potential public health effort to delay the onset of cognitive impairment and prevent the dementia pandemic in older people.

**Supplementary Information:**

The online version contains supplementary material available at 10.1186/s12877-022-03375-y.

## Background

Alzheimer’s disease and other related dementias (ADRD) have become a leading cause of disability and mortality in older adults worldwide [1, 2]. Given rapid population aging, the prevalence of ADRD is mounting on a global scale, especially in middle- and low-income countries with speedier aging [3, 4]. For example, China has the largest number of older adults with dementia in the world accounting for 25% of the worlds’ dementia cases and the annual incidence of dementia is more than 0.36 million [4]. In the absence of cure for the disease, dementia places a huge burden on families and health care systems as older adults gradually lose their health, independence, and ability to accomplish daily life [4]. Thus, preventing the progression of cognitive decline to dementia among older adults is critical to combat dementia epidemics [4, 5].

Studies have shown that environmental factors, such as air pollution [5–7] are important risk factors of cognitive decline. Emerging evidence also shows that drinking water quality could be a risk factor of dementia [8–11]. A variety of contaminants detected in drinking water, such as disinfection byproducts (DBPs), pharmaceutical and personal care products (PPCPs), endocrine-disrupting compounds (EDCs), antibiotic resistance genes, pathogens, microcystin, and toxins produced by cyanobacteria, have been found neurovirulent to people and to link to major neurogenerative diseases including dementia [9, 11, 12]. These contaminants are toxic to human body and may lead to DNA damage and death of brain cells, resulting in neurobehavioral impairment and cognitive dysfunction [8]. Additionally, exposure to polluted drinking water has been found to be associated with a higher risk of having dysfunctional cholesterol status and developing dyslipidemia, which is associated with brain aging and perhaps cognitive dysfunction [11, 13, 14].

Dyslipidemia refers to a group of metabolic derangements characterized by any or a combination of raised low-density lipoprotein (LDL), raised total cholesterol (TC), raised triglycerides (TG), and low high-density lipoprotein (HDL) [15]. It is well established that cholesterol is crucial in the development and functioning of the central nervous system [16]. Elevated serum cholesterol can promote the production and accumulation of beta-amyloid plaques in brain and brain ischemia resulting in dementia [16, 17]. Apolipoprotein E (APOE) and clusterin (CLU) involved in cholesterol transport and metabolism, are associated with an increased risk of Alzheimer’s disease (AD), indicating the underlying predictive roles of cholesterol metabolism in cognitive decline [18–21]. Additionally, plasma metabolite molecules distinguishing AD patients from controls is found to be mostly derived from cholesteryl esters (CE) [19, 20]. Collectively, these studies might support the hypothesis that dyslipidemia are present in cognitive decline.

Dyslipidemia is a major risk factor of cardiovascular disease [22] and a growing body of research suggests that cardiovascular risk factors and conditions are possible mediators of the association between adverse environmental exposure and cognitive decline [23–26]. Epidemiological evidence has shown that contaminants in drinking water can inhibit reverse cholesterol transport, increase in plasma free fatty acid, and constrain key regulators of lipid homeostasis, resulting in dyslipidemia [27]. Additionally, current research suggests that dysfunctional lipid parameters, such as high LDL-C or raised TG, is associated with cognitive decline [28–31] or lower domain-specific cognitive function in older adults [29, 30]. Therefore, it is reasonable to hypothesize that dyslipidemia might mediate the relationship between drinking water quality and cognitive function.

In China, the prevalence of dyslipidemia has exponentially risen from 18.6% to 40.4% over the past 10 years due to dramatic urbanization and socioeconomic transitions [13, 15]. With the rapid industrialization and economic development in China, environmental pollution has become a serious problem in the country [8]. Water pollution has been reported everywhere from surface water to groundwater and from freshwater to seawater [12]. Chronic exposure to various contaminants in the environment through inhalation of polluted air, consumption of contaminated water or food, or skin contact has shown to be related with adverse health outcomes including dyslipidemia and cognitive impairment and early death in Chinese [7, 8, 13]. However, environment-led neuro injury is largely understudied because of the lack of evidence with population data and the difficulties in finding the related environmental toxins [11, 32]. There is even less information on the relationship between drinking water quality and cognitive function for older adults in China and the population-based evidence is still unavailable. Although elevated LDL has been shown to be associated with cognitive decline [33, 34], the relationship between dyslipidemia and cognitive function has been less explored and results have been inconsistent [5, 14]. Some studies reported no association or an inverse association between TC and cognitive function in adults aged 65 and above [35, 36], whereas others showed a positive association in midlife with a longer follow-up observational period [5, 37]. Additionally, new lines of studies have suggested a positive association between water contaminants and the incidence of dyslipidemia in China [13, 38], however the evidence was limited to small provincial/state data, which hardly represents the population characteristics in China.

In light of high prevalence of dyslipidemia and dementia in China, it is critical to study how drinking water quality is associated with dyslipidemia and cognitive function and whether dyslipidemia mediates the relationship between drinking water quality and cognitive function. If drinking water affects cognitive function via dyslipidemia, the improvement of drinking water quality and correction of dyslipidemia may have considerable benefits for prevention of dementia in late adulthood and for the reduction of health care and financial burden. Therefore, the main aim of the current study was to explore the relationship between drinking water quality and cognitive function in adults aged 60 and above in China. We also hypothesized that the relationship could be mediated by dyslipidemia.

## Methods

### Data source

Data for this study were selected from the 2015 follow-up survey of China Health and Retirement Longitudinal Study (CHARLS). The national baseline survey of CHARLS was conducted between June 2011 and March 2012 including17,705 respondents from 10,257 households. Subsequential biennial follow-ups were conducted in 2013, 2015, and 2018.CHARLS interviews a nationally representative sample of adults 45 years and older, as well as their spouses when possible. The survey includes information on various variables at individual, household, and community levels. The sample was obtained through multistage cluster sampling, with an overall response rate of 80.5%, and nearly 64.2% in rural areas at the baseline survey [39, 40]. Data on drinking water quality were not available in the CHARLS so we used prefecture-level (city-level) water quality data from the Institute of Public and Environmental (IPE) Affairs in China (http://wwwen.ipe.org.cn/). The IPE data on drinking water quality were not collected until 2012 and only limited to major cities in China from 2012–2013. The nation-wide prefecture-level data on drinking water quality was collected in 2015 and beyond. Accordingly, we used the 2015 prefecture-level water quality data from the IPE and merged them with the 2015 CHARLS data by city names. We also collected prefecture-level data on gross domestic product (GDP) per capita from the China City Statistical Yearbook and other local yearbooks, and data on temperature and precipitation from the Physical Sciences Laboratory of the University of Delaware Air Temperature & Precipitation V5.01 (https://psl.noaa.gov/data/gridded/data.UDel_AirT_Precip.html). Air pollution data (measured by particulate matter ≤ 2.5 μm; PM_2.5_) were from the Atmospheric Composition Analysis.

Group [41]. Data on terrain/typography and community-level education came from the CHARLS community survey conducted in 2011/2012.

Our study sample included 5,749 respondents aged 60 years and above who responded the baseline survey in 2011/2012 and the 2015 follow-up survey. Among those, approximately 3% of cases were missing on cognitive function, 2% on self-reported dyslipidemia diagnosis, less than 1% on blood TG, and 8% on control variables due to item-nonresponse and lost to follow up in 2015. We deleted those missing cases and our final sample consisted of 4,951 observations.

### Measurement

#### Cognitive function

This study examined three composite measures of cognitive function: mental status, episodic memory, and global cognition. In CHARLS, mental status was assessed by two cognitive tests, the Telephone Interview of Cognitive Status (TICS) battery and figure drawing test [6]. The TICS test included ten items on orientation and numeric ability. Orientation was assessed by asking respondents to name today’s date (month, day, year), season, and identify the correct day of the week. Numeric ability was assessed through the serial sevens test, which asked respondents to subtract 7 from 100 (up to five times) and whether additional explanation or a piece of paper and a pencil was needed to complete the task. Correct answer to each item was 1 point and the total TICS score ranged from 0 to 10. Figure drawing test assessed participants’ visual and spatial abilities. Participants were asked to accurately re-draw a previously shown picture. Participants who successfully reproduced a similar picture received 1 point, and those who failed received 0 points. The final score of mental status was the sum scores from TICS and the figure drawing test, ranging from 0 to 11 [6]. Episodic memory was assessed through an immediate word recall based on respondents’ capacity to immediately repeat in any order ten Chinese nouns just read to them, followed by a delayed recall that tests respondents’ ability to repeat the same list of words 4 min later [42]. A single score of episodic memory was calculated by the sum of immediate and delayed recall scores, and it ranged from 0 to 20. Last, global cognition was the sum score from scores of mental status and episodic memory, ranging from 0 to 31. For all three measures, higher scores indicate better cognitive function [29].

#### Drinking water quality

Drinking water quality was a component of the Blue City Water Quality Index (BCWQI) developed by the IPE in China. The BCWQI is developed to score quality of surface water, groundwater, and drinking water based on the annual national water quality data issued by the Chinese government departments, which are collected by 600,000 water quality data points from 337 municipal cities and 25 counties every year [43]. In China, local ecology and environment departments and local water and natural resource departments provide the long-term monitoring of surface water and groundwater quality [43]. The BCWQI for scoring drinking water quality was primarily based on 2015 monitoring data for centralized drinking water sources (referring specifically to water bodies that support communities of 1,000 people or more, in both urban and rural areas) published by ecology and environment bureaus at the provincial/state, municipal and county level and above, combined with water source remediation progress reports [43]. Drinking water sources were comprehensively evaluated based on water quality classifications, transgressions of pollution standards and the progress of environmental rectifications [43]. Lower BCWQI scores indicated higher water quality, whereas higher BCWQI scores indicated lower water quality. Due to skewness of data distribution, we grouped the BCWQI scores into two groups based on the 2018 Blue City Water Quality Index report suggesting that scores less than 7.6 indicating *Excellent* or *Good* quality (coded 1), whereas scores of 7.6 and above indicating *Moderate*/*Relatively Poor*/*Poor* quality (coded 0) [43].

#### Dyslipidemia

Dyslipidemia was measured by lipid panel and self-reported dyslipidemia diagnosis. The two measures of dyslipidemia were used as two separate endogenous variables in our analytical models. Participants underwent at least 10-h overnight fasting before taking 8 ml venous blood samples by trained nurses in township hospitals or at a local office of China Center for Disease Prevention and Control (CDC) [44, 45]. Serum HDL, LDL, TC, and TG were measured by enzymatic colorimetric assays using an auto analyzer [44, 45]. Based on American Heart Association classification, dyslipidemia was defined as: HDL < 35 mg/dl, LDL > 130 mg/dl, TC > 200 mg/dl, or TG > 150 mg/dl, or a combination of them [46]. Blood TG was selected to be the biomarker measure of dyslipidemia in the current study because evidence from the CHARLS baseline data has shown that 15.2% of the middle aged and older population suffered from high levels of TG, which is a public health problem in China [44]. Moreover, our preliminary analyses showed that HDL, LDL, and TC were not correlated with our cognitive outcomes.

Self-reported dyslipidemia diagnosis was measured by a single survey question” *Have you been diagnosed with dyslipidemia by a doctor*? by the time of interview. Participants responded *yes* to the item, if they have been diagnosed with dyslipidemia by a doctor; and *no*, if they never have had a diagnosis. In the 2015 follow-up survey, blood collection occurred simultaneously with the interviews and the shipping temperature was strictly controlled and monitored by the CHARLS team [45].

##### *Covariates*

In the current study, we included individual- and community-level covariates. Individual-level covariates included age in years, gender (female versus male), residential area (urban versus rural), marital status (married versus unmarried), educational levels (primary school or less versus middle school and above), depressive symptoms [47], smoking status [5, 6], obesity [48], chronic diseases such as diabetes and cardiovascular disease [5, 6]. The measure of depressive symptoms was based on responses to the 10-item version of the Center for Epidemiologic Studies Short Depression Scale (CES-D 10). Each of the items had four response options coded from 0 to 3. The total score was the sum of points for all ten items. A dichotomous variable was created using a score of 10 or greater to indicate the presence of depressive symptoms [49]. Smoking status was measured as current use of tobacco (yes/no). Obesity was determined by body mass index (BMI). BMI was defined as weight in kilograms divided by height in meters squared, and BMI ≥ 28 was categorized as obese [6, 38]. To measure financial resources, we calculated annual household living expenditures. The existing literature has shown that household living expenditures are a better measure of economic resources available to the family than income in developing countries [50]. The summed total of annual household living expenditures was log-transformed. Chronic diseases were measured as self-reported diagnosis of diabetes or cardiovascular disease (yes/no).

Community-level covariates included community level of education, prefecture-level GDP per capita in Chinese *yuan*, annual total precipitation, average temperatures in January and July, annual average PM_2.5_, and terrain/topography [12]. In the CHARLS community survey, percentages of adult residents having completed different levels of education (i.e., illiterate/semi-illiterate, primary school, middle school, high school, college, and graduate school) within each community were collected. Based on the percentage of each level of education, we calculated the percentage of high school or above education within each community and constructed a proxy variable using that percentage to measure community-level education. A higher percentage indicated a higher level of education within a community. Prefecture-level GDP per capita was log-transformed to correct skewness. The weather data from Udel-AirT_Precip V5.01 were mainly drawn from the Global Historical Climatology Network and Legates and Willmott’s station records of monthly and annual mean air temperature and total precipitation, which provided monthly time series of surface air temperature and precipitation with approximately 50 km*50 km resolution [51]. We applied ArcGIS zonal statistics to extract annual average PM_2.5_, monthly temperatures, and annual precipitations for each prefecture. PM_2.5_ refers to atmospheric particulate matter (PM) that has a diameter of < 2.5 µm and PM_2.5_ level and has been found to be an important factor associated with individual cognitive function [23, 24, 52, 53]. China regional PM_2.5_ (V4.CH.03) was estimated by combining Aerosol Optical Depth (AOD) retrievals from the NASA’s Medium Resolution Imaging Spectroradiometer (MODIS), Multiangle Imaging Spectroradiometer (MISR), and Sea-Viewing Wide Field-of-View Sensor (SeaWiFS) satellite instruments and coincident aerosol vertical profiles with the GEOSChem chemical transport model, and subsequently calibrated to regional ground-based observations using geographically weighted regression (GWR) and it provided annual time series of PM_2.5_ concentration with approximately 1 km × 1 km resolution [54–56]. We used the average annual PM_2.5_ from 2000 to 2010 to capture the possibly delayed effect of air pollution on cognitive function [57]. The temperatures in January and July and annual precipitation were the average for 1981 to 2010 and were dichotomized to capture extreme weather conditions: annual total precipitation (> = 800 mm), temperatures in January (< -10^◦^C/14^◦^F) and July (≥ 28^◦^C/82.4^◦^F) [38]. Topography has been shown a key factor associated with water quality [58, 59]. Different landscape will influence the temperature, pH scale, deposition, and pollution of water and all of those affect drinking water quality [58, 59]. Terrain/topography was measured by one item in the CHARLS community survey: *What is the main terrain/topography of your village /community*? with 5 response categories including *plain*, *hill*, *plateau*, *mountainous region*, and *basin*. We recoded this variable dichotomously as *plain* vs. *other*s.

### Statistical analysis

First, we utilized mixed-effects models with maximum likelihood estimation to estimate the fixed and random effect coefficients and variances of drinking water quality and dyslipidemia measures on each measure of cognitive function, respectively. Next, path analyses with maximum likelihood estimation were conducted to examine whether the impact of drinking water quality on each measure of cognitive function was mediated by dyslipidemia. The mixed effects models are appropriate to assess the impacts of drinking water quality and dyslipidemia on cognitive function when the variables included in the models occur at different levels [39]. In our study, two-level linear mixed models were used with individuals as our level-1 variable and communities as the level-2 variable. In our models, the intercept(s) (grand mean scores of cognitive function across individuals and communities) and slopes (relationships of drinking water quality, dyslipidemia, and cognitive function across individuals and communities) were specified as random at level 2. We incorporated random intercepts for the cluster variable ‘terrains’. This accounted for the correlation of respondents within terrains.

Path analyses were performed to address the hypothesized pathways between drinking water quality and each measure of cognitive function through self-reported dyslipidemia diagnosis or blood TG after controlling for individual- and community-levelcovariates. The effects estimated in each path analysis were determined using two ANCOVA models (12 ANCOVA models in total). In the first ANCOVA model, the direct effect of drinking water quality on cognitive function was determined based on estimates from a model adjusting for the correlations between exogenous variables at individual and community levels. The second ANCOVA model examined the relationship between drinking water quality and dyslipidemia (self-reported dyslipidemia or blood TG), while adjusting the impacts of individual- and community-level covariates. The indirect effect of drinking water quality, passing through dyslipidemia, on cognitive function was calculated by multiplying the coefficient estimate from the first model with the estimates from the second model. The total effect is the sum of the direct and indirect effects. We checked the normality of scores of mental status, episodic memory, and global cognition using one-factor ANOVAs, and 95% confidence intervals (CIs) were used. Errors associated with scores of cognitive functions were found to be normally distributed. All analyses and procedures were conducted in SAS, version 9.4 (SAS Institute, Inc., Cary, North Carolina). Mixed effects models used the PROC MIXED procedure and path analyses used the PROC CALIS procedure.

Considering attrition from the baseline to the follow-up survey and missing data on study variables that did not occur at random, we also used multiple imputations with multivariate normal distribution to replace those missing cases and adjust for this potential bias in the analyses (see Supplementary Materials for the results). The results were not substantially different from those without multiple imputations.

## Results

Table 1 showed that the mean age of the participants was 68 years old (*SD* = 6.6) including 65.6% rural residents. Nearly 50.1% of participants were women and 84.3% were married. The average educational level was low with approximately 80.37% having a primary school or less education. Individuals with high levels of blood TG (> 150 mg/dl) accounted for 29.6% and those who reported that they have had dyslipidemia diagnosis by a doctor accounted for 32.2%. There were 69.9% of individuals living in areas with an BCWQI score less than 7.6 for drinking water, indicating either *Excellent* or *Good* quality. The average scores of cognitive measures were 12.4 (*SD* = 6.0, range 0–31) for global cognition, 6.9 for mental status (*SD* = 3.3, range 0–11), and 5.5 for episodic memory (*SD* = 3.6, range 0–20). For individual health status, 20.3% of participants reported having diabetes and 35.5% having cardiovascular disease. The obesity rate was relatively low at 11.1% and 28.3% of respondents had depressive symptoms. As for smoking status, 43.6% were current smokers. For community characteristics, the mean percentage of high school or above education at community level was 21.7% (*SD* = 13.9%, range 0–95%). The log of prefecture-level GDP per capita was 10.25. Approximately 13.3% of areas had an average January temperature < -10 °C/14°F, 20.4% had an average July temperature >  = 28 °C/82.4°F, and 59.9% had annual total precipitation >  = 800 mm. The average annual PM_2.5_ concentration from 2000 to 2010 was 46.88 μg/m^3^ (*SD* = 18.8). As for geological terrain, 42.2% were plain.

**Table 1:** Descriptive Characteristics of the Study Using CHARLS 2015 (*N* = 4,951)

	*M* or *%*	*SD*
**Drinking Water Quality**		
*Excellent/Good* (BCWQI scores < 7.6)	69.87	
*Moderate/Relatively Poor/Poor* (BCWQI scores > = 7.6)	30.13	
**Having self-reported dyslipidemia**	32.21	
**Blood TG (**> 150 mg/dl)	29.61	
**Cognitive function, scores**		
Mental status (0–11)	6.91	3.34
Episodic memory (0–20)	5.48	3.63
Global cognition (0–31)	12.40	6.04
***Individual level covariates***		
Age, years	68.09	6.56
Female	50.39	
Urban residence	34.41	
Annual household living expenditure (ln)	9.12	2.14
Middle-school-or-above education	19.63	
Married	84.29	
Current cigarette smoker ^a^	43.61	
Having depressive symptoms	28.28	
Having diabetic symptoms	20.30	
Having cardiovascular diseases	35.53	
Obesity (BMI > = 28 kg/m^2^)	11.14	
***Community-level covariates***		
Prefecture GDP per capita (ln)	10.25	0.55
Community level of education (percentage of high school or above education)	21.70	13.88
Annual total precipitation (> = 800 mm)	59.87	
Annual temperature in January (< -10^◦^C/14^◦^F)	13.27	
Annual temperature in July (≥ 28^◦^C/82.4^◦^F)	20.40	
Plain terrain	42.15	
Ten-year ^b^ average PM_2.5_ concentration (μg/m^3^, 1kmx1km spatial resolution)	46.88	18.79

Note. Numeric variables presented as mean (*SD*) and categorical variables presented as counts (%). *SD* standard deviation; *M* mean

^a^Never smokers and past smokers combined as the reference group

^b^Annual average PM_2.5_ concentration from 2000–2010

Tables 2, 3 and 4 presented the results from the mixed effects models. Table 2 examined the associations between drinking water quality and cognitive function including mental status, episodic memory, and global cognition. Exposure to high quality drinking water was significantly associated with higher scores in mental status, episodic memory, and global cognition (β = 0.32, *p* < 0.001 for mental status; β = 0.24, *p* < 0.05 for episodic memory; β = 0.53, *p* < 0.01 for global cognition). Tables 3 and 4 examined the associations between self-reported dyslipidemia diagnosis or blood TG and the three cognitive measures. In Table 3, respondents who reported dyslipidemia diagnosis had higher scores in the three composite measures of cognitive function (β = 0.39, *p* < 0.001 for mental status; β = 0.27 *p* < 0.05 for episodic memory; β = 0.65, *p* < 0.001 for global cognition). In Table 4, an elevated TG cholesterol was associated with higher scores in mental status (β = 0.21, *p* < 0.05), but not with episodic memory or global cognition. However, the impacts of drinking water quality and dyslipidemia (i.e., self-reported dyslipidemia or TG cholesterol) on cognitive function did not vary by terrains.

**Table 2:** Associations between Drinking Water Quality and Cognitive Function from Mixed Effects Models, CHARLS 2015 (*N* = 4,951)

	Mental Status	Episodic Memory	Global Cognition
**Fixed effects**			
Intercept	4.38***(0.97)	10.95***(1.13)	24.41***(1.07)
Drinking water quality (ref. BCWQI scores > = 7.6)	0.34***(0.09)	0.24*(0.11)	0.58**(0.17)
**Individual-level controls**			
Age (years)	-0.08***(0.01)	-0.14***(0.01)	-0.22***(0.01)
Female (ref. male)	-1.87***(0.08)	-0.14(0.10)	-2.02***(0.15)
Urban (ref. rural)	0.76***(0.09)	0.65***(0.10)	1.40***(0.16)
Annual household living expenditure (ln)	0.14***(0.02)	0.13***(0.02)	0.27***(0.04)
Education (ref. primary school or below)	1.86***(0.11)	1.97***(0.12)	3.83***(0.19)
Married (ref. not married)	0.20(0.13)	0.06(0.15)	0.28(0.23)
Current cigarette smokers	-0.11(0.08)	-0.08(0.09)	-0.20(0.15)
Having depressive symptoms	-0.82***(0.09)	-0.87***(0.11)	-1.69***(0.16)
Having diabetes	0.10(0.10)	0.08(0.12)	0.19(0.18)
Having cardiovascular disease	0.26**(0.09)	0.13(0.10)	0.40**(0.15)
Obesity (ref. BMI < 28 km/m^2^)	0.19(0.13)	0.07(0.15)	0.24(0.23)
**Community-level controls**			
Prefecture GDP per capita (ln)	0.63***(0.08)	0.24**(0.09)	0.94***(0.14)
Community level of education (percentage of high school or above education)	0.01***(0.01)	0.01*(0.01)	0.02***(0.01)
Annual total precipitation (> = 800 mm)	-0.02(0.10)	-0.51***(0.12)	-0.51**(0.18)
Annual temperature in January (< -10^◦^C/14^◦^F)	0.10(0.16)	0.39*(0.18)	0.48*(0.28)
Annual temperature in July (≥ 28^◦^C/82.4^◦^F)	-0.28*(0.11)	-0.18(0.13)	-0.47*(0.20)
Ten-year ^a^ average PM_2.5_ concentration (μg/m^3^, 1kmx1km spatial resolution)	-0.01(0.01)	-0.01(0.01)	-0.01(0.01)
**Random effects**			
Terrains (*plain* vs. *others*)	0.02(0.02)	0.04(0.04)	0.10(0.12)
Residual	7.76*** (0.16)	10.43***(0.21)	24.89***(0.50)

*Note*. Values are based on SAS Proc Mixed and expressed as parameter estimates *β* (standard errors). Estimation method = ML (maximum likelihood); Satterthwaite degrees of freedom

^*^p < .05, ** *p* < .01, *** *p* < .001

a. Annual average PM_2.5_ concentration from 2000–2010

**Table 3:** Associations between Self-Reported Dyslipidemia Diagnosis and Cognitive Function from Mixed Effects Models, CHARLS 2015 (*N* = 4,951)

	Mental Status	Episodic Memory	Global Cognition
**Fixed effects**			
Intercept	4.93***(0.95)	11.49***(1.11)	16.46***(1.72)
Self-reported Dyslipidemia	0.39***(0.09)	0.27*(0.11)	0.66***(0.17)
**Individual-level controls**			
Age (years)	-0.08***(0.01)	-0.14***(0.01)	-0.22***(0.01)
Female (ref. male)	-1.88***(0.08)	-0.15(0.10)	-2.03***(0.15)
Urban (ref. rural)	0.75***(0.09)	0.63***(0.10)	1.37***(0.16)
Annual household living expenditure (ln)	0.14***(0.02)	0.13***(0.02)	0.27***(0.04)
Education (ref. primary school or below)	1.82***(0.11)	1.96***(0.12)	3.77***(0.19)
Married (ref. not married)	0.13(0.13)	-0.01(0.15)	0.15(0.23)
Current cigarette smokers	-0.10(0.08)	-0.07(0.10)	-0.17(0.15)
Having depressive symptoms	-0.81***(0.09)	-0.87***(0.11)	-1.67***(0.16)
Having diabetes	-0.01(0.10)	-0.01(0.12)	-0.02(0.18)
Having cardiovascular disease	0.18*(0.09)	0.07(0.10)	0.26(0.16)
Obesity (ref. BMI < 28 km/m^2^)	0.16(0.13)	0.04(0.15)	0.19(0.23)
**Community-level controls**			
Prefecture GDP per capita (ln)	0.63***(0.08)	0.19*(0.09)	0.85***(0.14)
Community level of education (percentage of high school or above education)	0.01***(0.01)	0.01(0.00)	0.02***(0.01)
Annual total precipitation (> = 800 mm)	0.11(0.10)	-0.43***(0.11)	-0.31(0.18)
Annual temperature in January (< -10^◦^C/14^◦^F)	0.17(0.16)	0.41*(0.18)	0.57*(0.28)
Annual temperature in July (≥ 28^◦^C/82.4^◦^F)	-0.30**(0.11)	-0.21(0.13)	-0.53**(0.20)
Ten-year ^a^ average PM_2.5_ concentration (μg/m^3^, 1kmx1km spatial resolution)	-0.01(0.01)	-0.01(0.01)	-0.01(0.01)
**Random effects**			
Terrains (*plain* vs. *others*)	0.01(0.01)	0.03(0.04)	0.08(0.10)
Residual	7.68*** (0.15)	10.45***(0.21)	24.85***(0.50)

*Note*. Values are based on SAS Proc Mixed and expressed as parameter estimates *β* (standard errors). Estimation method = ML (maximum likelihood); Satterthwaite degrees of freedom

^*^p < .05, ** *p* < .01, *** *p* < .001

^a^Annual average PM_2.5_ concentration from 2000–2010

**Table 4:** Associations between TG Cholesterol and Cognitive Function from Mixed Effects Models, CHARLS 2015 (*N* = 4,951)

	Mental Status	Episodic Memory	Global Cognition
**Fixed effects**			
Intercept	4.98***(0.95)	11.42***(1.11)	16.44***(1.72)
Blood TG (ref. < = 150 mg/dl)	0.21*(0.09)	0.10(0.10)	0.30(0.16)
**Individual-level controls**			
Age (years)	-0.08***(0.01)	-0.14***(0.01)	-0.22***(0.01)
Female (ref. male)	-1.90***(0.09)	-0.15(0.10)	-2.06***(0.15)
Urban (ref. rural)	0.76***(0.09)	0.64***(0.10)	1.40***(0.16)
Annual household living expenditure (ln)	0.14***(0.02)	0.13***(0.02)	0.27***(0.04)
Education (ref. primary school or below)	1.85***(0.11)	1.97***(0.12)	3.81***(0.19)
Married (ref. not married)	0.21(0.13)	0.06(0.15)	0.29(0.23)
Current cigarette smokers	-0.10(0.08)	-0.08(0.10)	-0.18(0.15)
Having depressive symptoms	-0.81***(0.09)	-0.86***(0.11)	-1.67***(0.16)
Having diabetes	0.09(0.10)	0.07(0.12)	0.16(0.18)
Having cardiovascular disease	0.26**(0.09)	0.14(0.10)	0.40**(0.15)
Obesity (ref. BMI < 28 km/m^2^)	0.12(0.13)	0.04(0.15)	0.16(0.23)
**Community-level controls**			
Prefecture GDP per capita (ln)	0.62***(0.08)	0.22***(0.09)	0.84***(0.14)
Community level of education (percentage of high school or above education)	0.01***(0.01)	0.01(0.00)	0.02**(0.01)
Annual total precipitation (> = 800 mm)	0.10(0.10)	-0.40***(0.11)	-0.30(0.18)
Annual temperature in January (< -10^◦^C/14^◦^F)	0.18(0.13)	0.48***(0.15)	0.69**(0.24)
Annual temperature in July (≥ 28^◦^C/82.4^◦^F)	-0.31**(0.11)	-0.20(0.13)	-0.56**(0.20)
Ten-year ^a^ average PM_2.5_ concentration (μg/m^3^, 1kmx1km spatial resolution)	-0.01(0.01)	-0.01(0.01)	-0.01(0.01)
**Random effects**			
Terrains (*plain* vs. *others*)	0.02(0.02)	0.03(0.04)	0.12(0.13)
Residual	7.77*** (0.16)	10.43***(0.21)	25.00***(0.50)

*Note*. Values are based on SAS Proc Mixed and expressed as parameter estimates *β* (standard errors). Estimation method = ML (maximum likelihood); Satterthwaite degrees of freedom

^*^p < .05, ** *p* < .01, *** *p* < .001

^a^Annual average PM_2.5_ concentration from 2000–2010

**Fig. 1:**
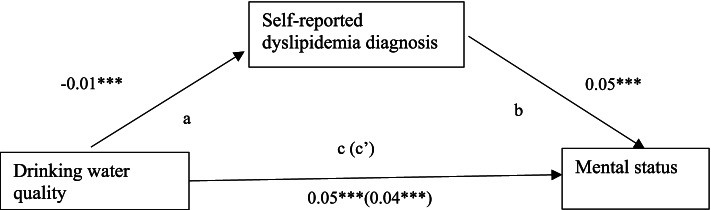
Hypothesized mediation model of relationships among drinking water quality, self-reported dyslipidemia, and mental status after controlling for individual and community covariates. Standardized path coefficients were presented. a. Relationship between drinking water quality and self-reported dyslipidemia. b. Association between self-reported dyslipidemia and mental status. c. Direct effect of drinking water quality on mental status. c’. Total effect of drinking water quality on mental status through self-reported dyslipidemia. *p < 0.05, **p < 0.01, ***p < 0.001

**Fig. 2:**
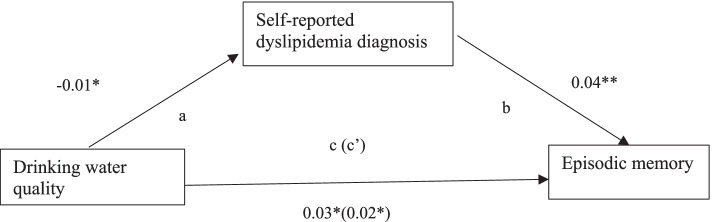
Hypothesized mediation model of relationships among drinking water quality, self-reported dyslipidemia, and episodic memory after controlling for individual and community covariates. Standardized path coefficients were presented. a. Relationship between drinking water quality and self-reported dyslipidemia. b. Association between self-reported dyslipidemia and episodic memory. c. Direct effect of drinking water quality on episodic memory. c’. Total effect of drinking water quality on episodic memory through self-reported dyslipidemia. *p < 0.05, **p < 0.01, ***p < 0.001

**Fig. 3:**
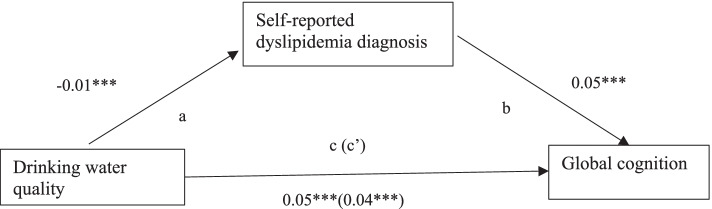
Hypothesized mediation model of relationships among drinking water quality, self-reported dyslipidemia, and global cognition after controlling for individual and community covariates. Standardized path coefficients were presented. a. Relationship between drinking water quality and self-reported dyslipidemia. b. Association between self-reported dyslipidemia and global cognition. c. Direct effect of drinking water quality on global cognition. c’. Total effect of drinking water quality on global cognition through self-reported dyslipidemia. *p < 0.05, **p < 0.01, ***p < 0.001

Direct, indirect, and total effects for the hypothesized mediation models were presented in Figs. 1, 2 and 3. As shown in Fig. 1, the direct effect of drinking water quality on mental status was 0.05 (*p* < 0.001) indicating that good drinking water quality predicted higher scores of mental status. The indirect effect of drinking water quality through self-reported dyslipidemia diagnosis on mental status was negative (β = -0.01, *p* < 0.001) and the total effect of drinking water quality on mental status was 0.04 (*p* < 0.001). This indicates suppression, which means that the presence of dyslipidemia in the model increased the magnitude of the direct effect of drinking water quality on mental status. In Fig. 2, the suppression effect of self-reported dyslipidemia diagnosis was also found for episodic memory, although the suppression effect was not as strong as for mental status. The direct effect of drinking water quality on episodic memory was 0.03 (*p* = 0.02) indicating that high quality drinking water predicted higher scores of episodic memory. The indirect effect of drinking water quality through self-reported dyslipidemia diagnosis on episodic memory was negative (β = -0.01, *p* = 0.01) and the total effect of drinking water quality on episodic memory was 0.02 (*p* = 0.04). Self-reported dyslipidemia diagnosis also increased the effect of drinking water quality on global cognition rather than decreasing it suggesting a suppression effect. In Fig. 3, the direct effect of drinking water quality on global cognition was 0.05 (*p* < 0.001) indicating that high quality drinking water predicted higher scores of global cognition. The indirect effect of drinking water quality through self-reported dyslipidemia diagnosis on global cognition was negative (β = -0.01, *p* < 0.001) and the total effect of drinking water quality on global cognition was 0.04 (*p* < 0.001). The indirect effects of drinking water quality through blood TG on mental status or global cognition were not significant indicating there was no mediation effect of blood TG in the relationship between drinking water quality and cognitive function. No significant relationships were observed for episodic memory.

## Discussion

To our best knowledge, this is the first study to examine the associations among drinking water quality, dyslipidemia, and cognitive function for older adults in China. Using the mixed effects models, we found that exposure to high quality drinking water was associated with better cognitive function in older Chinese and the relationship was independent of natural or socioeconomic variations in neighborhood environments. This finding is consistent with previous work suggesting a positive association between drinking water quality and cognitive function using either Chinese or Western samples [8, 9, 11]. Despite cross-sectional evidence, this result is significant in identifying and confirming the environmental risk factor that affected cognitive function in older adults using a nationally representative sample. Improving drinking water quality can be a potential public health effort to delay the onset of cognitive impairment and prevent the dementia pandemic in China.

Using the path analyses, we also investigated the pathways from drinking water quality to cognitive function through the presence of dyslipidemia. Our finding suggests that high quality drinking water was associated with a lower risk of having dyslipidemia diagnosis in late adulthood after controlling for individual characteristics and neighborhood environments including community level of education, terrain, PM_2.5_, annual precipitation and temperature, and neighborhood GDP. This finding was in agreement with previous epidemiological studies that used state- or community-level data to find the detrimental impact of polluted water on blood lipoprotein [13, 38]. Our result contributed to the literature in providing population-based evidence to confirm that drinking water quality can be a modifiable risk factor of dyslipidemia among older adults.

Moreover, self-reported dyslipidemia diagnosis was found to be a suppressor in the relationship between drinking water quality and cognitive function as the association between drinking water quality and cognition is stronger after controlling for dyslipidemia status. This finding added new evidence to the existing literature in identifying the suppression effect of dyslipidemia in the pathway from drinking water quality to cognitive function, thus provided the basis for future research to identify the mechanism of the process. Our study suggests that this suppression effect is largely due to the positive association between the presence of dyslipidemia measured by either self-reported data or blood TG and cognitive performance. These findings were somewhat surprising, but they are consistent with some prior studies which reported an inverse association between dementia and triglyceride levels in the short-term observational period after lipid measurement or measurement in later life (age ≥ 65) [5, 35]. As the direct transport of lipid in plasma takes longer time to pass through the blood–brain barrier to arrive the brain, thus plasma levels might not reflect the lipid levels in the brain over a short period of time and longer periods might be required to observe clinically evident cognitive decline [5]. Our findings contribute to the existing literature by providing new evidence based on both self-reported and biomarker measures of dyslipidemia in an older population and highlighting an area that warrants further investigation in the future.

However, our study has serval limitations. First, drinking water information was not collected by CHARLS and our alternative data from IPE were not collected until 2015, thus our study does not capture changes in drinking water quality or track changes in cognitive function. Our cross-sectional evidence cannot ascertain a causal relationship between drinking water quality and cognitive function or explicate the mechanism of how dyslipidemia suppressed the predictor power of drinking water quality on cognitive function. Existing literature suggests that the onset of cognitive decline led by polluted water or dyslipidemia could take more than 10-year follow-up duration [60], we will further verify our results in the future work using longer-term panel data. Second, modest selection bias might exist, as older adults who have undergone cognitive decline may not be as likely to respond to the survey interview. Our estimated associations might not be applied to those not selected into our cohort. Third, although we adjusted for many known risk factors for dyslipidemia and cognitive function, we cannot exclude the possibility of unmeasured confounding factors. For example, whether or not older adults used lipid-lowering drugs to control for their blood cholesterol, lifestyle risk including diet, or contaminants in drinking water were not adjusted in our analyses, as the information is not available in CHARLS. Physical activity is another important factor associated with cognitive function in older adults [61, 62]. However, only random sub-sample (half) of households were selected to respond to the survey items on physical activity in CHARLS. Therefore, half of respondents in our analytical sample did not have data on physical activity. Although we applied multiple imputation to address this issue (see Supplementary Materials), this sampling issue on the measure of physical activity in CHARLS might affect the external validity of our results. Thus, we did not include physical activity in our analytical model. Last, lipid measurements were not taken routinely or at multiple timepoints during the observational period, thus we assessed the association between dyslipidemia measured at a single timepoint and cognitive decline risk, which is likely to be weaker than associations assessed for an average level over multiple timepoints of the risk factor because of regression dilution bias [5, 63].

Despite these limitations, the findings of our study add new knowledge to the growing literature on environmental risk factors of cognitive health. Given the rising prevalence of dementia in China, if high quality drinking water is positive on cognitive function among older adults, these findings could be used as evidence to advance policy, practice, and research in preventing and/or delaying the onset of cognitive impairment. Our findings provide implications for policymakers in China to revise the existing safe drinking water policy to strengthen monitoring and detecting drinking water quality, especially areas where drinking water was polluted. Policies are needed to allocate more financial and technological resources to control water pollution and provide safe and healthy drinking water to the affected neighborhoods. Health care policies are needed to require neurocognitive screening tests and assessment as a part of routine exams at primary care settings for older adults, especially those who live in areas with low quality drinking water, as early detection is vital in preventing or postponing the onset of dementia in older adults [64].

Despite relatively small correlation coefficients between drinking water quality and dyslipidemia, our study provides important information to health care professionals about the relationship between environment and chronic diseases and help them identify cardiovascular and/or behavioral risk of cognitive decline, which would inspire creation of cognitive-behavioral interventions to encourage drinking safe and healthy water and the use of lipid-lowering drugs to control blood cholesterol, given the high prevalence of dyslipidemia in China. Moreover, public health education including programs to improve awareness of the effects of drinking water on health outcomes including cognitive function for the general population should be created and delivered to communities, especially those suffering from water pollution. If these preventive efforts begin earlier, we may be able prevent the dementia epidemic and optimize quality of life for the aging population.

## Conclusion

Our study suggested that high quality drinking water was associated with better cognitive function among older adults in China, and the presence of dyslipidemia magnified the positive associations between drinking water quality and cognitive function. Although the causal association between drinking water quality and cognitive function cannot be defined using the cross-sectional data, our results suggest drinking water quality could be a risk factor of cognitive impairment, which can be affected by blood cholesterol levels in late adulthood.

## Supplementary Information

**Additional file 1: Supplementary Table 1.** Associations between Drinking Water Quality and Cognitive Function from Mixed Effects Models, CHARLS 2015 Imputed Data (*N* = 5,749). **Supplementary Table 2.** Associations between Self-Reported Dyslipidemia Diagnosis and Cognitive Function from Mixed Effects Models, CHARLS 2015 Imputed Data (*N* = 5,749). **Supplementary Table 3.** Associations between TG Cholesterol and Cognitive Function from Mixed Effects Models, CHARLS 2015 Imputed Data (*N* = 5,749). **Supplementary Figure 1.** Hypothesized mediation model of relationships among drinking water quality, self-reported dyslipidemia, and mental status after controlling for individual and community covariates using the imputed data. Standardized path coefficients were presented. **Supplementary Figure 2.** Hypothesized mediation model of relationships among drinking water quality, self-reported dyslipidemia, and episodic memory after controlling for individual and community covariates using the imputed data. Standardized path coefficients were presented. **Supplementary Figure 3.** Hypothesized mediation model of relationships among drinking water quality, self-reported dyslipidemia, and global cognition after controlling for individual and community covariates using the imputed data. Standardized path coefficients were presented.

## Data Availability

The datasets generated and analyzed during the current study are not publicly available due to concerns of outsourcing and arbitrary use of data but are available from the corresponding author on reasonable request.

## Abbreviations

CHARLS: China Health and Retirement Longitudinal Study
ADRD: Alzheimer’s disease and related dementias
AD: Alzheimer’s disease
BMI: Body mass index
DBPs: Disinfection byproducts
PPCPs: Pharmaceutical and personal care products
EDCs: Endocrine-disrupting compounds
LDL: Low-density lipoprotein
TC: Total cholesterol
TG: Triglycerides
HDL: High-density lipoprotein
GDP: Gross domestic product
TICS: The Telephone Interview of Cognitive Status
IPE: The Institute of Public and Environmental Affairs
BCWQI: The Blue City Water Quality Index
CES-D 10: 10-Item Center for Epidemiological Studies Depression Scale
ANCOVA: Analysis of covariance
SD: Standard deviation
IC: Confidence interval
ML: Maximum likelihood
M: Mean
PM: Particulate matter
AOD: Aerosol optical depth
MODIS: Medium resolution imaging spectroradiometer
MISR: Multiangle imaging spectroradiometer
SeaWiFS: Sea-viewing wide field-of-view sensor
APOE: Apolipoprotein E
CLU: Clusterin
CE: Cholesteryl esters
GWR: Geographically weighted regression
