# Supplementary material for: Associations among drinking water quality, dyslipidemia, and cognitive function for older adults in China: evidence from CHARLS
Source: BMC Geriatr. 2022 Aug 18;22:683. doi: 10.1186/s12877-022-03375-y (PMC9386986; doi:10.1186/s12877-022-03375-y)
Supplement: Supplementary file 1 — Additional file 1: Supplementary Table 1. Associations between Drinking Water Quality and Cognitive Function from Mixed Effects Models, CHARLS 2015 Imputed Data (N = 5,749). Supplementary Table 2. Associations between Self-Reported Dyslipidemia Diagnosis and Cognitive Function from Mixed Effects Models, CHARLS 2015 Imputed Data (N = 5,749). Supplementary Table 3. Associations between TG Cholesterol and Cognitive Function from Mixed Effects Models, CHARLS 2015 Imputed Data (N = 5,749). Supplementary Figure 1. Hypothesized mediation model of relationships among drinking water quality, self-reported dyslipidemia, and mental status after controlling for individual and community covariates using the imputed data. Standardized path coefficients were presented. Supplementary Figure 2. Hypothesized mediation model of relationships among drinking water quality, self-reported dyslipidemia, and episodic memory after controlling for individual and community covariates using the imputed data. Standardized path coefficients were presented. Supplementary Figure 3. Hypothesized mediation model of relationships among drinking water quality, self-reported dyslipidemia, and global cognition after controlling for individual and community covariates using the imputed data. Standardized path coefficients were presented. [file 12877_2022_3375_MOESM1_ESM.docx]

**Supplementary Materials**

We included physical activity as a covariate in the mixed-effects models and path analyses using the imputed data. The CHARLS asked about three categories of physical activities: (i) vigorous physical activities that required hard/high-intensity physical effort, (ii) moderate physical activities that required a moderate physical effort, and (iii) walking at home or at work for travel, recreation, or leisure. For each category of physical activities, the respondents were asked whether they performed these activities for at least 10 minutes continuously in a usual week, the number of days they performed them, and how much time they spent on those days. Based on this information, we calculated the total number of hours a respondent spent on physical activities in a normal week. To obtain a normal distribution, the log form of the variable was calculated and analyzed. Results from the mixed-effects models are demonstrated in Supplementary Tables 1-3 and results from the path analyses are shown in Supplementary Figures 1-3.

Supplementary Table 1. Associations between Drinking Water Quality and Cognitive Function from Mixed Effects Models, CHARLS 2015 Imputed Data (N = 5,749)

|  | Mental Status | Episodic Memory | Global Cognition |
| --- | --- | --- | --- |
| **Fixed effects** |  |  |  |
| Intercept | 4.52***(0.98) | 10.39***(1.14) | 14.91***(1.80) |
| Drinking water quality (ref. BCWQI scores >= 7.6) | 0.27**(0.09) | 0.17(0.10) | 0.44*(0.16) |
| **Individual-level controls** |  |  |  |
| Age (years) | -0.08***(0.01) | -0.14***(0.01) | -0.23***(0.01) |
| Female (ref. male) | -1.85***(0.08) | -0.15(0.09) | -1.99***(0.14) |
| Urban (ref. rural) | 0.73***(0.09) | 0.61***(0.10) | 1.34***(0.16) |
| Annual household living expenditure (ln) | 0.14***(0.02) | 0.14***(0.02) | 0.28***(0.04) |
| Education (ref. primary school or below) | 1.85***(0.10) | 2.00***(0.12) | 3.85***(0.18) |
| Married (ref. not married) | 0.16(0.11) | 0.11(0.13) | 0.27(0.23) |
| Current cigarette smokers | -0.23*(0.08) | -0.14(0.09) | -0.37*(0.14) |
| Physical exercise (hours/week; ln) | 0.01(0.01) | 0.01(0.01) | 0.01(0.01) |
| Having depressive symptoms | -0.85***(0.09) | -0.88***(0.10) | -1.73***(0.16) |
| Having diabetes | 0.12(0.10) | 0.08(0.12) | 0.21(0.17) |
| Having cardiovascular disease | 0.23*(0.09) | 0.13(0.10) | 0.36*(0.15) |
| Obesity (ref. BMI<28km/m^2^) | 0.27*(0.12) | 0.10(0.14) | 0.37(0.22) |
| **Community-level controls** |  |  |  |
| Prefecture GDP per capita (ln) | 0.69***(0.07) | 0.29**(0.09) | 0.97***(0.14) |
| Community level of education (*percentage of* *high school or above*) | 0.02***(0.01) | 0.01**(0.01) | 0.03***(0.01) |
| Annual total precipitation (>=800mm) | -0.04(0.10) | -0.57***(0.11) | -0.61**(0.17) |
| Annual temperature in January (<-10^◦^C/14^◦^F) | 0.18(0.15) | 0.52**(0.17) | 0.69*(0.27) |
| Annual temperature in July (≥28^◦^C/82.4^◦^F) | -0.18(0.10) | -0.07(0.11) | -0.24(0.18) |
| Ten-year ^a^ average PM_2.5_ concentration (μg/m^3^, 1kmx1km spatial resolution) | -0.01(0.01) | -0.01(0.01) | -0.01(0.01) |
| **Random effects** |  |  |  |
| Terrain (plain vs. others) | 0.02(0.02) | 0.04(0.04) | 0.12(0.13) |
| Residual | 7.76*** (0.16) | 10.43***(0.21) | 24.98***(0.50) |

*Note*. Values are based on SAS Proc Mixed and expressed as parameter estimates *β* (standard errors). Estimation method = ML (maximum likelihood); Satterthwaite degrees of freedom.

* *p* < .05, ** *p* < .01, *** *p* < .001

a. Annual average PM_2.5_ concentration from 2000-2010.

Supplementary Table 2. Associations between Self-Reported Dyslipidemia Diagnosis and Cognitive Function from Mixed Effects Models, CHARLS 2015 Imputed Data (N = 5,749)

|  | Mental Status | Episodic Memory | Global Cognition |
| --- | --- | --- | --- |
| **Fixed effects** |  |  |  |
| Intercept | 5.11***(0.97) | 10.91***(1.14) | 16.02***(1.79) |
| Self-reported Dyslipidemia | 0.37***(0.09) | 0.28*(0.10) | 0.65***(0.16) |
| **Individual-level controls** |  |  |  |
| Age (years) | -0.08***(0.01) | -0.14***(0.01) | -0.22***(0.01) |
| Female (ref. male) | -1.85***(0.08) | -0.15(0.10) | -2.00***(0.14) |
| Urban (ref. rural) | 0.70***(0.09) | 0.60***(0.10) | 1.29***(0.16) |
| Annual household living expenditure (ln) | 0.14***(0.02) | 0.13***(0.02) | 0.27***(0.04) |
| Education (ref. primary school or below) | 1.82***(0.11) | 1.98***(0.12) | 3.77***(0.19) |
| Married (ref. not married) | 0.10(0.11) | 0.03(0.13) | 0.13(0.20) |
| Current cigarette smokers | -0.10(0.08) | -0.13(0.09) | -0.34*(0.14) |
| Physical activity (hours/week, ln) | 0.01(0.01) | 0.01(0.01) | 0.01(0.01) |
| Having depressive symptoms | -0.86***(0.09) | -0.89***(0.10) | -1.75***(0.16) |
| Having diabetes | 0.02(0.10) | 0.02(0.11) | 0.05(0.18) |
| Having cardiovascular disease | 0.17(0.09) | 0.08(0.10) | 0.26(0.16) |
| Obesity (ref. BMI<28km/m^2^) | 0.22(0.12) | 0.07(0.14) | 0.29(0.22) |
| **Community-level controls** |  |  |  |
| Prefecture GDP per capita (ln) | 0.64***(0.07) | 0.25**(0.09) | 0.89***(0.13) |
| Community level of education (*percentage of* *high school or above*) | 0.01***(0.01) | 0.01*(0.01) | 0.02***(0.01) |
| Annual total precipitation (>=800mm) | 0.07(0.09) | -0.51***(0.11) | -0.44*(0.17) |
| Annual temperature in January (<-10^◦^C/14^◦^F) | 0.21(0.15) | 0.51**(0.17) | 0.72*(0.27) |
| Annual temperature in July (≥28^◦^C/82.4^◦^F) | -0.21*(0.10) | -0.10(0.12) | -0.32(0.18) |
| Ten-year ^a^ average PM_2.5_ concentration (μg/m^3^, 1kmx1km spatial resolution) | -0.01(0.01) | -0.01(0.01) | -0.01(0.01) |
| **Random effects** |  |  |  |
| Terrain (plain vs. others) | 0.01(0.02) | 0.03(0.04) | 0.09(0.11) |
| Residual | 7.71*** (0.16) | 10.46***(0.21) | 24.93***(0.50) |

*Note*. Values are based on SAS Proc Mixed and expressed as parameter estimates *β* (standard errors). Estimation method = ML (maximum likelihood); Satterthwaite degrees of freedom.

* *p* < .05, ** *p* < .01, *** *p* < .001

a. Annual average PM_2.5_ concentration from 2000-2010.

Supplementary Table 3. Associations between TG Cholesterol and Cognitive Function from Mixed Effects Models, CHARLS 2015 Imputed Data (N = 5,749)

|  | Mental Status | Episodic Memory | Global Cognition |
| --- | --- | --- | --- |
| **Fixed effects** |  |  |  |
| Intercept | 4.98***(0.97) | 10.71***(1.13) | 15.70***(1.78) |
| Blood TG (ref. <= 150mg/dl) | 0.16(0.09) | 0.10(0.10) | 0.26(0.15) |
| **Individual-level controls** |  |  |  |
| Age (years) | -0.08***(0.01) | -0.14***(0.01) | -0.23***(0.01) |
| Female (ref. male) | -1.87***(0.08) | -0.16(0.10) | -2.04***(0.14) |
| Urban (ref. rural) | 0.73***(0.09) | 0.61***(0.10) | 1.34***(0.16) |
| Annual household living expenditure (ln) | 0.14***(0.02) | 0.14***(0.02) | 0.29***(0.04) |
| Education (ref. primary school or below) | 1.84***(0.10) | 1.97***(0.12) | 3.83***(0.18) |
| Married (ref. not married) | 0.15(0.11) | 0.11(0.13) | 0.27(0.20) |
| Current cigarette smokers | -0.22*(0.08) | -0.14(0.09) | -0.36*(0.14) |
| Physical activity (hours/week, ln) | 0.01(0.01) | 0.01(0.01) | 0.01(0.01) |
| Having depressive symptoms | -0.83***(0.09) | -0.88***(0.10) | -1.71***(0.16) |
| Having diabetes | 0.10(0.10) | 0.09(0.11) | 0.20(0.18) |
| Having cardiovascular disease | 0.23*(0.09) | 0.14(0.10) | 0.37*(0.15) |
| Obesity (ref. BMI<28km/m^2^) | 0.22(0.12) | 0.04(0.15) | 0.30(0.22) |
| **Community-level controls** |  |  |  |
| Prefecture GDP per capita (ln) | 0.64***(0.07) | 0.25**(0.09) | 0.89***(0.13) |
| Community level of education (*percentage of* *high school or above*) | 0.02***(0.01) | 0.01**(0.01) | 0.03***(0.01) |
| Annual total precipitation (>=800mm) | 0.06(0.09) | -0.50***(0.11) | -0.44*(0.17) |
| Annual temperature in January (<-10^◦^C/14^◦^F) | 0.22(0.15) | 0.54**(0.17) | 0.75*(0.27) |
| Annual temperature in July (≥28^◦^C/82.4^◦^F) | -0.19(0.10) | -0.07(0.12) | -0.26(0.18) |
| Ten-year ^a^ average PM_2.5_ concentration (μg/m^3^, 1kmx1km spatial resolution) | -0.01(0.01) | -0.01(0.01) | -0.01(0.01) |
| **Random effects** |  |  |  |
| Terrain (plain vs. others) | 0.02(0.02) | 0.03(0.04) | 0.12(0.13) |
| Residual | 7.77*** (0.16) | 10.45***(0.21) | 25.00***(0.50) |

*Note*. Values are based on SAS Proc Mixed and expressed as parameter estimates *β* (standard errors). Estimation method = ML (maximum likelihood); Satterthwaite degrees of freedom.

* *p* < .05, ** *p* < .01, *** *p* < .001

a. Annual average PM_2.5_ concentration from 2000-2010.

Self-reported dyslipidemia diagnosis

0.05***

-0.01***

a

b

c (c’)

Mental status

Drinking water quality

0.04***(0.03***)

Supplementary Figure 1. Hypothesized mediation model of relationships among drinking water quality, self-reported dyslipidemia, and mental status after controlling for individual and community covariates using the imputed data. Standardized path coefficients were presented.

a. Relationship between drinking water quality and self-reported dyslipidemia

b. Association between self-reported dyslipidemia and mental status

c. Direct effect of drinking water quality on mental status

c’. Total effect of drinking water quality on mental status through self-reported dyslipidemia.

**p*< 0.05, ***p*< 0.01, ****p*<0.001.

Episodic memory

Self-reported dyslipidemia diagnosis

0.03***

c (c’)

b

-0.003***

a

Drinking water quality

0.03***(0.02***)

Supplementary Figure 2. Hypothesized mediation model of relationships among drinking water quality, self-reported dyslipidemia, and episodic memory after controlling for individual and community covariates using the imputed data. Standardized path coefficients were presented.

a. Relationship between drinking water quality and self-reported dyslipidemia

b. Association between self-reported dyslipidemia and episodic memory

c. Direct effect of drinking water quality on episodic memory

c’. Total effect of drinking water quality on episodic memory through self-reported dyslipidemia.

**p*< 0.05, ***p*< 0.01, ****p*<0.001.

Self-reported dyslipidemia diagnosis

-0.01***

0.05***

b

a

c (c’)

Drinking water quality

0.04***(0.03***)

Global cognition

Supplementary Figure 3. Hypothesized mediation model of relationships among drinking water quality, self-reported dyslipidemia, and global cognition after controlling for individual and community covariates using the imputed data. Standardized path coefficients were presented.

a. Relationship between drinking water quality and self-reported dyslipidemia

b. Association between self-reported dyslipidemia and global cognition

c. Direct effect of drinking water quality on global cognition

c’. Total effect of drinking water quality on global cognition through self-reported dyslipidemia.

**p*< 0.05, ***p*< 0.01, ****p*<0.001.
